# Supplementary description of *Boulenophrys elongata* (Anura, Megophryidae): Sexual dimorphism and observations on reproductive behaviours

**DOI:** 10.3897/BDJ.14.e201422

**Published:** 2026-07-23

**Authors:** Jian Wang, Bin-Bin Zhan, Lin Tan, Wei-Wen Xiao, Yuan-Hang Li, Shi-Shi Lin, Zhao-Chi Zeng

**Affiliations:** 1 Guangdong Polytechnic of Environmental Protection Engineering, Foshan, China Guangdong Polytechnic of Environmental Protection Engineering Foshan China; 2 Guangdong Wildlife Monitoring Rescue and Conservation Center, Guangzhou, China Guangdong Wildlife Monitoring Rescue and Conservation Center Guangzhou China

**Keywords:** ecology, morphology, montane amphibian, pre-amplexus behaviour, taxonomy

## Abstract

**Background:**

The genus *Boulenophrys* displays high species diversity, yet most species are known only from isolated localities with narrow geographic distributions. Fundamental ecological data and reproductive traits remain largely undocumented across the genus. *Boulenophrys elongata* is one of the least investigated species in this group. Given that the male type specimens of this species exhibited incompletely developed breeding-related secondary sexual characteristics, the sexual dimorphism of the species has remained poorly clarified.

**New information:**

We conducted systematic field surveys at the type locality of *Boulenophrys elongata* and collected five adult individuals during the breeding season, enabling supplementation of morphological diagnoses and baseline ecological data. Through long-term in situ monitoring, we recorded the full sequence of pre-amplexus reproductive behaviour, constituting the first formal documentation of such behaviour in the genus. This study addresses critical gaps in the biological understanding of *B. elongata* and the genus and provides empirical support for population assessments and conservation strategies for montane amphibians.

## Introduction

The genus *Boulenophrys* Fei, Ye & Jiang, 2016 (Fei and Ye 2016) represents a highly diverse clade of Asian horned toads, with a current global diversity of 84 recognised species (Frost 2026, Wang et al. 2026). This genus is predominantly distributed across East and Southeast Asia, with its core diversity concentrated in southern China, extending southwards into northern Vietnam, northern Laos, north-eastern Myanmar and northern Thailand (Fei and Ye 2016, Lyu et al. 2023). Many species are characterised by narrow geographic ranges, high levels of endemism and limited dispersal capacity, which are closely linked to complex topography and habitat fragmentation across southern China and adjacent regions (Wang et al. 2019, Wang et al. 2022, Liu et al. 2018, Lyu et al. 2023, Zhao et al. 2025). Despite recent advances in systematic and molecular studies, a substantial proportion of *Boulenophrys* species remain poorly documented, with critical gaps in basic biological data, especially regarding sexual dimorphism, habitat use and reproductive ecology (Lin et al. 2024, Zeng et al. 2024).

*Boulenophrys elongata* Zeng, Wang, Chen, Xiao, Zhan, Li and Lin, 2024 was described from eastern Guangdong, China, in 2024 (Zeng et al. 2024). To date, this species is known exclusively from its type locality, representing another narrow-range endemic species within the genus. Notably, the original male type specimens exhibited under-developed secondary sexual characteristics related to reproduction. With merely two male and three female specimens available, sexual dimorphism of this species remains insufficiently clarified. Furthermore, ecological observations and reproductive data for *B. elongata* (also for the genus) are entirely absent, limiting comprehensive understanding of its natural history and conservation status. To address these knowledge gaps, we conducted repeated systematic field surveys and long-term monitoring at the type locality, collected breeding individuals and documented the first observations of pre-amplexus reproductive behaviour in this species. The present study provides supplementary morphological data, clarifies sexual dimorphism and reports detailed ecological and reproductive information for *B. elongata*, representing the first formal record of reproductive behaviour in the genus *Boulenophrys*.

## Materials and methods

### Morphology

Five newly-collected specimens of *Boulenophrys elongata* from the type locality were examined in this study, including three adult males and two adult females. All specimens examined were fixed in 10% buffered formalin and later transferred to 70% ethanol. All studied specimens have been deposited at the Guangdong Polytechnic of Environmental Protection Engineering (GEP), in Foshan, Guangdong Province.

External measurements were recorded with a digital caliper (Neiko 01407A stainless steel 6-inch digital caliper) to the nearest 0.1 mm. These measurements are as follows: ED (eye diameter, from the anterior corner of the eye to posterior corner of the eye); FTL (foot length, from distal end of shank to the tip of digit IV); HDL (head length, from tip of snout to the articulation of the jaw); HDW (head width, head width at the commissure of the jaws); HND (hand length, from the proximal border of the outer palmar tubercle to the tip of digit III); IND (internasal distance, distance between nares); IOD (interorbital distance, minimum distance between upper eyelids); RAD (forearm or radiulna length, from the flexed elbow to the proximal border of the outer palmar tubercle); SNT (snout length, from tip of snout to the anterior corner of the eye); SVL (snout-vent length, from tip of snout to posterior margin of vent); TD (tympanum diameter, horizontal diameter of tympanum); TED (tympanum-eye distance, from anterior edge of tympanum to posterior corner of the eye); TIB (crus or tibiofibula length, from the outer surface of the flexed knee to the heel). Sex was determined by external secondary sexual characters, such as the presence of vocal sacs, nuptial pads or nuptial spines in males and their absence in females (Fei et al. 2009).

### Ecological and Behavioural Observations

Systematic field surveys and continuous in situ monitoring were conducted at the type locality of *Boulenophrys elongata* (Mt. Lianhua, Huidong County, Guangdong) throughout 2024 and 2025 and in 5/2026. Surveys were carried out diurnally and nocturnally until male advertisement calls were detected, covering both non-breeding and breeding periods. All fieldwork strictly followed low‑disturbance protocols to minimise anthropogenic interference with their natural behaviour.

Behavioural observations were performed via non‑invasive focal animal sampling. Upon detection of individuals, observers maintained a minimum distance of 3–5 m and remained stationary for a 15‑minute acclimatisation period to allow resumption of natural behaviour. Low‑intensity yellow light (8 lux) was used for visual observations and video recording, avoiding bright white light that could disrupt natural activity and communication. All behavioural sequences were recorded in high‑definition video (1080 p, 30 fps) and annotated in real time to document activity patterns, social interactions and reproductive behaviour.

Reproductive phenology was determined by integrating year‑round survey data, detection of male calling activity and observations of reproductive traits. Pre‑amplexus reproductive behaviour was monitored continuously under undisturbed natural conditions, with the duration of each behavioural phase quantified to the nearest second. No physical contact, handling or habitat modification was conducted during behavioural monitoring to preserve natural breeding sequences.

## Taxon treatments

### Boulenophrys elongata

Zeng, Wang, Chen, Xiao, Zhan, Li & Lin, 2024

89799A94-EC2E-5761-B7B0-827C8132E630

#### Materials

- **Type status:** Other material. **Occurrence:** catalogNumber: GEP a434; recordedBy: Jian Wang; individualCount: 1; sex: male; lifeStage: adult; occurrenceID: 59FD5746-3C4D-5121-A5E0-E30566809030; **Taxon:** scientificName: *Boulenophrys elongata*; **Location:** country: China; stateProvince: Guangdong; county: Huidong; locality: Mt Lianhua; verbatimElevation: 940 m; decimalLatitude: 23.065212; decimalLongitude: 115.240888; georeferenceProtocol: GPS; **Identification:** identifiedBy: Jian Wang; dateIdentified: 2026; **Event:** eventDate: 9/5/2025; **Record Level:** language: en; collectionCode: Amphibians; basisOfRecord: PreservedSpecimen
- **Type status:** Other material. **Occurrence:** catalogNumber: GEP a435; recordedBy: Jian Wang; individualCount: 1; sex: female; lifeStage: adult; occurrenceID: 7ADF338D-7625-5AFF-A6BA-20BE91E3D8EA; **Taxon:** scientificName: *Boulenophrys elongata*; **Location:** country: China; stateProvince: Guangdong; county: Huidong; locality: Mt Lianhua; verbatimElevation: 940 m; decimalLatitude: 23.065212; decimalLongitude: 115.240888; georeferenceProtocol: GPS; **Identification:** identifiedBy: Jian Wang; dateIdentified: 2026; **Event:** eventDate: 9/5/2025; **Record Level:** language: en; collectionCode: Amphibians; basisOfRecord: PreservedSpecimen
- **Type status:** Other material. **Occurrence:** catalogNumber: GEP a436; recordedBy: Jian Wang; individualCount: 1; sex: female; lifeStage: adult; occurrenceID: 98C9441E-1660-50C7-A29C-F9E55F1D0277; **Taxon:** scientificName: *Boulenophrys elongata*; **Location:** country: China; stateProvince: Guangdong; county: Huidong; locality: Mt Lianhua; verbatimElevation: 940 m; decimalLatitude: 23.065212; decimalLongitude: 115.240888; georeferenceProtocol: GPS; **Identification:** identifiedBy: Jian Wang; dateIdentified: 2026; **Event:** eventDate: 9/5/2025; **Record Level:** language: en; collectionCode: Amphibians; basisOfRecord: PreservedSpecimen
- **Type status:** Other material. **Occurrence:** catalogNumber: GEP a557; recordedBy: Jian Wang; individualCount: 1; sex: male; lifeStage: adult; occurrenceID: 44F8408B-89E1-5D85-81FE-FA0A0AFF2FC2; **Taxon:** scientificName: *Boulenophrys elongata*; **Location:** country: China; stateProvince: Guangdong; county: Huidong; locality: Mt Lianhua; verbatimElevation: 940 m; decimalLatitude: 23.065212; decimalLongitude: 115.240888; georeferenceProtocol: GPS; **Identification:** identifiedBy: Jian Wang; dateIdentified: 2026; **Event:** eventDate: 29/03/2026; **Record Level:** language: en; collectionCode: Amphibians; basisOfRecord: PreservedSpecimen
- **Type status:** Other material. **Occurrence:** catalogNumber: GEP a558; recordedBy: Jian Wang; individualCount: 1; sex: male; lifeStage: adult; occurrenceID: 5C809D3B-D747-5CF2-AF63-C0E7F13DF240; **Taxon:** scientificName: *Boulenophrys elongata*; **Location:** country: China; stateProvince: Guangdong; county: Huidong; locality: Mt Lianhua; verbatimElevation: 940 m; decimalLatitude: 23.065212; decimalLongitude: 115.240888; georeferenceProtocol: GPS; **Identification:** identifiedBy: Jian Wang; dateIdentified: 2026; **Event:** eventDate: 29/3/2026; **Record Level:** language: en; collectionCode: Amphibians; basisOfRecord: PreservedSpecimen

#### Revised Diagnoses

**Figure 1.**
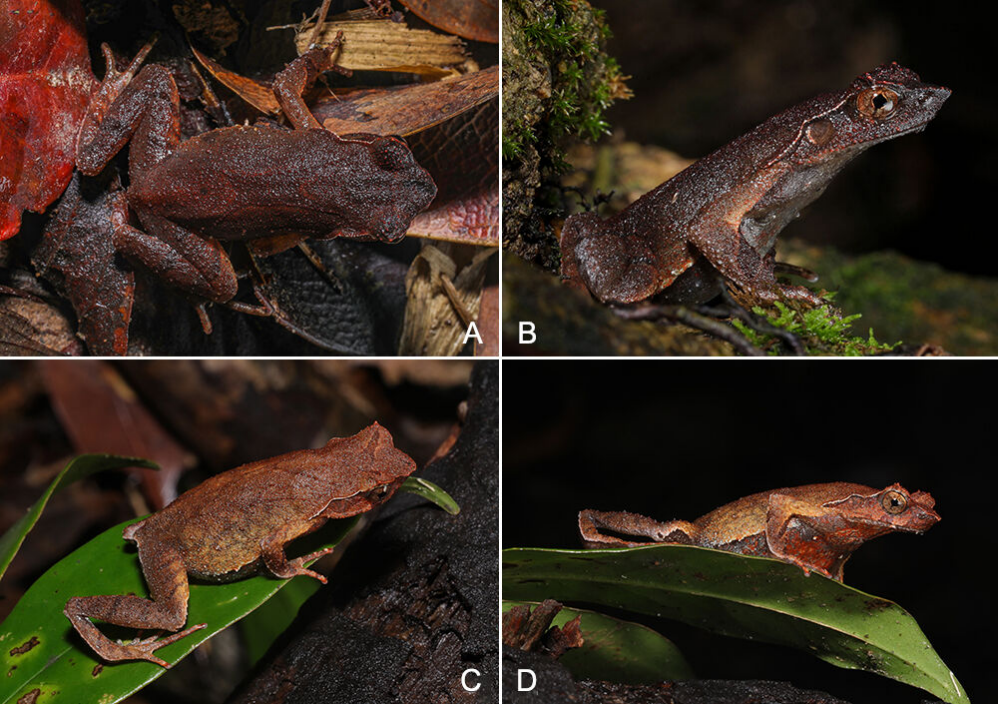
Life aspect of *Boulenophrys elongata*: **A, B** Dorsal and lateral views of the male specimen GEP a434; **C, D** Dorsolateral and lateral views of the female specimen GEP a435.

(1) small size (SVL 27.8–30.2 mm in five adult males, SVL 34.6–37.6 mm in five adult females), see Table 1 for detailed measurements; (2) canthus rostralis well developed, tongue not notched posteriorly; (3) tympanum and tympanic margin distinct; (4) vomerine ridges and vomerine teeth present; (5) dorsal skin relatively smooth with dense granules, weak discontinuous X-shaped ridge on centre of dorsum, dorsolateral ridges absent, sparse large tubercles on flanks, dorsal limbs with discontinuous transverse ridges and tubercles, ventral skin smooth; (6) outer margin of upper eyelid with a small horn-like prominent tubercle, supratympanic fold distinct and narrow, curving posteroventrally to above arm; (7) two metacarpal tubercles distinct, inner one observably enlarged; relative finger lengths I < II < IV < III; distinct subarticular tubercle at base of each finger; (8) body and limbs slender, heels just meeting when hind-limbs folded; tibiotarsal articulation reaching the region from posterior corner to middle of eye; (9) toes without webbing and lateral fringes, inner metatarsal tubercle long ovoid, outer one absent; (10) dorsal surface dark brown to orange brown with irregular dark-brown patches and dark-brown triangular marking between eyes, dorsal limbs and digits light brown with dark brown transverse bands; (11) a nuptial pad with dense black nuptial spines at the bases of fingers I and II respectively **in breeding males**; (12) subgular vocal sac present **in males**; (13) dense tiny conical spines present on dorsum of **breeding males**. Life-related manifestations are illustrated in Fig. 1.

#### Ecological Traits

**Figure 2.**
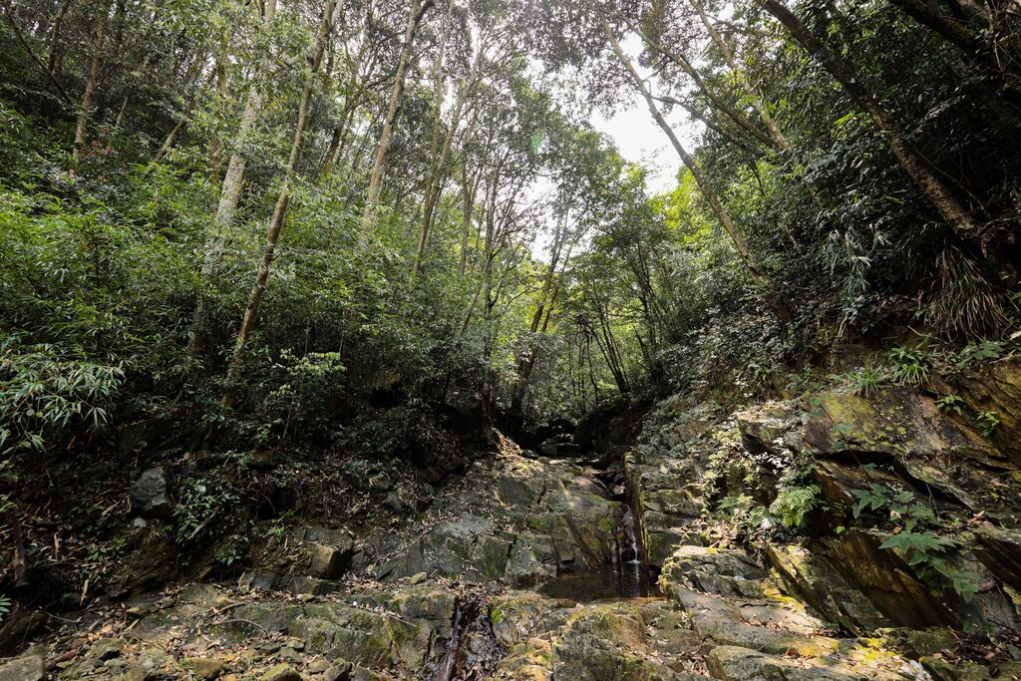
Habitat of *Boulenophrys elongata* in Mt. Lianhua of Huidong County, Huizhou City, Guangdong, China.

*Boulenophrys elongata* is known from Mt. Lianhua (700–1000 m a.s.l.) of Huidong County, Huizhou City, Guangdong, China. This toad inhabits flowing montane streams and the nearby forest floor in subtropical evergreen broad-leaved forest (Fig. 2). As stated in Zeng et al. (2024), no individuals were encountered during field surveys conducted in 2, 5, 9, 10/2023. Male advertisement calls were recorded in 6, 7/2023. Nevertheless, the under-developed translucent nuptial pads and nuptial spines in males, along with immature eggs in females, indicated that the breeding season of *Boulenophrys elongata* had not yet commenced at that time. However, reproductive behaviour of this species was documented in our field surveys conducted in early 5/2025 and late 3/2026. Males collected in early 5/2025 and late 3/2026 exhibited nuptial pads and spines, whereas females collected in early 5/2025 carried mature eggs. Integrating all available survey data, the breeding season may span from early March to late August. Its reproductive activities, represented by male calling behaviour, exclusively occur during or immediately after heavy rainfall. In addition, male advertisement calls can no longer be heard if rainfall ceases for several successive days, even under relatively high ambient humidity.

#### Reproductive Behaviour

**Figure 3.**
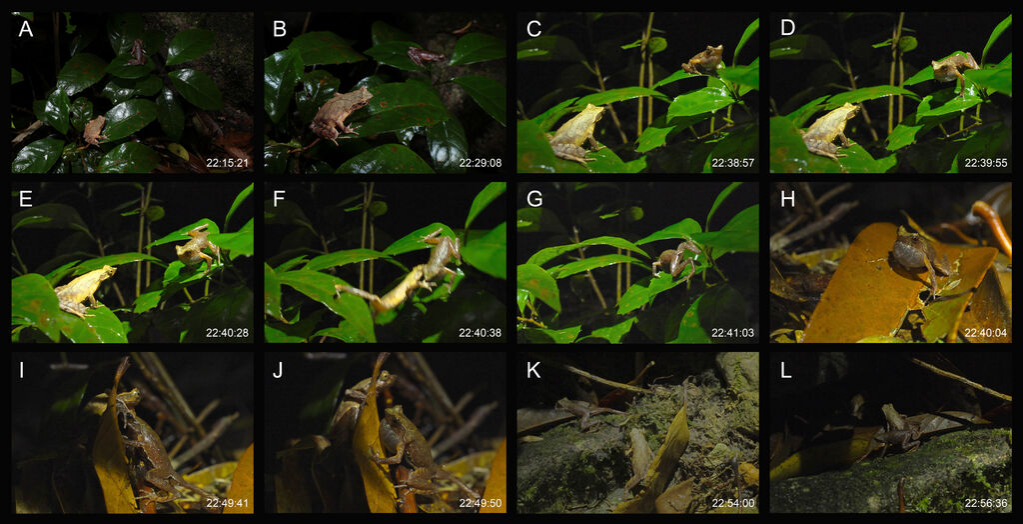
Pre-amplexus behaviour of *Boulenophrys elongata*: A male emits advertisement calls to lure a female on to riparian shrub leaves (**A**); The male re-orientates its body and resumes calling (**B**); The male initially detects the approaching female (**C**); The male leans forward and keeps vocalising (**D**); The male leans forward to produce calls again (**E**); The female makes a forward leap and collides with the male, representing the first physical interaction (**F**); After the female drops into leaf litter, the male adjusts posture and peers downwards to search for her (**G**); The male utters calls after jumping on to ground litter (**H**); The male moves close to the female and establishes the second physical contact (**I**); The male resumes vocalisation subsequent to the second physical contact (**J**); The male guides the female to move in the direction of his advertisement calls (**K**); The male steps across the female’s back, prior to amplexus (**L**).

Continuous observation on the pre-amplexus courtship behaviour of *Boulenophrys elongata* was carried out for 56 minutes with minimal field disturbance on 28/3/2026. See Suppl. material 1 for the condensed process video and Fig. 3 for the flow diagram.

Males established calling territories on riparian shrub foliage at around 25 cm above the ground. They produced intermittent quack-like advertisement calls via a single subgular vocal sac to attract conspecific females and defend their home ranges.

Under dim light conditions, the male called intermittently, while the female stretched its body forward on leaves. The male ceased calling after 13 seconds, presumably affected by light and resumed vocalisation 1 minute and 54 seconds later. Just 4 seconds into the new calling bout, the female rapidly adjusted its body orientation twice and crawled one step towards the male during the second adjustment. At 22:36:36, the male detected the female and stopped calling immediately. Two seconds later, it emitted lower-frequency and lower-pitch calls for 5 consecutive seconds, with a straight-line distance of approximately 8 cm separating the two individuals.

The male kept calling intermittently and the female slowly approached and observed the calling direction. The male positioned its body sideways facing the female and maintained vocal activity. After 6 seconds of calling, the female adjusted posture swiftly, blinked once and faced the male directly, prompting the male to halt calling instantly. Thirty-two seconds afterwards, the male called again for 8.5 seconds. At the 8^th^ second of this period, the female blinked and slightly turned its head towards the male, leading to immediate termination of vocalisation. The male then slowly turned to face the female, crawled 1.5 cm forward and blinked once and called for another 10 seconds. Receiving no response, it moved an additional 1 cm and blinked twice and restarted calling.

In the 2^nd^ second of renewed calling, the female lifted its head and blinked simultaneously to face the male. This subtle movement interrupted the call, yet the male soon resumed calling at a slightly lower frequency. Five seconds later, the female blinked again and tilted its head about 15 degrees sideways. The male perceived this movement and further reduced its calling frequency, with the inter-individual distance narrowed to 4.5 cm. Two seconds later, the female launched a short forward jump with fore-limbs spread out. Snout and fore-limb collision occurred, but the female failed to land on the male and fell 25 cm down into leaf litter.

Ten seconds after the fall, the male leaned towards the female’s location, adjusted its posture and blinked five times (the female was out of sight at this moment) and continued calling at the original frequency. The female made three unsuccessful attempts to climb and jump back on to the foliage. Seven minutes later, the male jumped down to the ground litter and resumed calling. After 6 seconds of vocalisation, the female adjusted posture with eye blinking. The male stopped calling and crawled slowly towards the female, then paused, blinked once and called for 5 seconds. The female adjusted its body posture twice accompanied by blinking during this period and the male moved closer to establish the second physical contact, with continuous blinking in response throughout the process.

The male first touched the posterior margin of the female’s right snout with its snout tip, then rubbed the third and fourth digits of the female’s right fore-limb inwards and outwards using its snout and eyelids for 10 seconds before detachment. Following a 6-second call from the male, the female blinked, adjusted posture and made a short jump towards the male. The male turned around and called for 5 seconds. The female adjusted her body posture to face the male. After detecting the female’s responsive motion, the male began to crawl slowly in the direction of the advertisement calls.

The male switched to companion-leading courtship rather than actively searching for the female via vocalisation. This behavioural shift was recorded at 22:50:59, 14 minutes and 23 seconds after the female was first detected. The male crawled steadily towards the direction of the advertisement calls, while the female followed by alternating crawling and jumping; the maximum distance between the two individuals reached 60 cm. Seven intermittent physical contacts occurred throughout this movement. Both individuals exhibited blinking behaviour during locomotion and when changing travel directions. In cases where the two became separated, the male produced calls to re-orientate the female and the female performed a single blink immediately after relocating the male. From 23:11:31 to 23:11:36, the female jumped forwards along the route and the male moved over her back without initiating amplexus. The male only emitted calls when it lost sight of the female and resumed moving once the follower was visible again. The pair proceeded towards a seepage area concealed within rock crevices, approximately 12 m away. Observations ended at 23:11:30. The microhabitat here consisted of intricate narrow rock formations and dense shrubs, so monitoring was halted to prevent human disturbance to the natural courtship process. The total observation duration from the initial detection of the female was 34 minutes and 54 seconds.

A rival male produced continuous intermittent background calls throughout observation, but the focal female showed no behavioural distraction triggered by competing acoustic signals. No amplexus was observed during the whole monitoring process.

The entire pre-copulatory courtship process of *Boulenophrys elongata* in this single event can be categorised into five sequential behavioural stages. First, territorial males perch on riparian foliage and emit intermittent advertisement calls to attract conspecific females and defend their microhabitat territories. Second, females locate calling males through acoustic signals, conduct close-range mate evaluation and display frequent blinking behaviour during the assessment process. Third, mutual behavioural recognition and positional adjustment occur between males and females, with reciprocal blinking responses serving as typical interactive cues. Fourth, bilateral physical contact occurs after which males deliver tactile stimulation, blinking and vocal signals co-occurring during the whole tactile interaction process. Fifth, males guide females towards potential rocky seepage breeding microhabitats via sustained vocalisation and rhythmic blinking behaviour.

## Discussion

This study documents the first pre‑amplexus reproductive behaviour for *Boulenophrys* species, providing novel baseline data on montane horned toad mating strategies. The observed sequence of male calling, female assessment, snout contact and male‑led guiding suggests complex reproductive behaviour, providing preliminary insight into anuran adaptations in fragmented montane habitats. Limitations include interrupted observations due to dense vegetation and rugged terrain, restricted quantification under low‑light low‑disturbance conditions and a single recorded event. Repeated observations are needed to confirm the consistency and generality of these behavioural patterns across individuals and breeding seasons. Notably, sympatric distribution is widespread amongst *Boulenophrys* species (Liu et al. 2018, Lyu et al. 2023). Males of sympatric congeners exhibit obvious microhabitat partitioning for courtship activities (Qian et al. 2024). Species characterised by shorter limbs generally emit advertisement calls on the ground or within rock crevices (Wang et al. 2019). In view of the interspecific divergence in courtship microhabitats and behavioural traits across the genus, extensive and sustained field investigations are still required to comprehensively unravel the diversified reproductive behaviour of *Boulenophrys*.

For *Boulenophrys elongata*, this work resolves taxonomic ambiguity by describing breeding‑related sexual dimorphism absent in the original type series, refining morphological diagnosis. Ecological and behavioural data establish a baseline for conservation assessment and population monitoring. At the genus level, this finding provides a comparative framework for behavioural evolution and phylogenetic studies, highlighting the value of targeted fieldwork. Beyond the genus, this study confirms that sustained field surveys and long‑term monitoring are essential for effective anuran conservation.

## Supplementary Material

XML Treatment for Boulenophrys elongata

152DB845-949C-5744-9C61-FB2FF797D1ED 10.3897/BDJ.14.e201422.suppl1 Supplementary material 1 Pre-amplexus behaviors of *Boulenophrys elongata* Data type multimedia File: oo_1658847.mp4 https://binary.pensoft.net/file/1658847 Wang J, Zhan BB, Tan L, Xiao WW, Li YH, Lin SS, Zeng ZC

## Figures and Tables

**Table 1.** Measurements (in mm) of voucher specimens of *Boulenophrys elongata* examined in this study, * holotype, ^#^ paratype.

Voucher	GEP a150#	GEP a155*	GEP a434	GEP a557	GEP a558	n = 5
SEX	M	M	M	M	M	Males
SVL	28.2	28.5	27.8	27.9	30.2	27.8–30.2 (28.5 ± 1.0)
HDL	9.7	9.9	9.4	10.1	10.3	9.4–10.3 (9.9 ± 0.4)
HDW	9.1	9.2	9.3	9.9	9.9	9.1–9.9 (9.5 ± 0.4)
ED	3.9	3.7	3.6	3.5	3.3	3.3–3.9 (3.6 ± 0.2)
TD	2.1	1.9	1.8	1.8	2.1	1.8–2.1 (1.9 ± 0.1)
TED	0.9	1.1	1.1	1.2	1.2	0.9–1.2 (1.1 ± 0.1)
SNT	3.5	3.5	3.5	4.0	4.0	3.5–4.0 (3.7 ± 0.3)
IND	3.3	3.2	3.6	3.3	3.2	3.2–3.6 (3.3 ± 0.2)
IOD	3.1	3.0	3.5	3.2	3.1	3.0–3.5 (3.2 ± 0.2)
HDN	7.5	7.0	7.0	7.2	8.2	7.0–8.2 (7.4 ± 0.5)
RAD	6.0	5.7	6.2	5.5	6.4	5.5–6.4 (5.9 ± 0.4)
FTL	18.2	16.3	17.3	16.2	19.0	16.2–19.0 (17.4 ± 1.2)
TIB	12.4	12.0	12.1	11.9	13.6	11.9–13.6 (12.4 ± 0.7)
Voucher	GEP a151#	GEP a152#	GEP a156#	GEP a435	GEP a436	n = 5
SEX	F	F	F	F	F	Females
SVL	35.1	35.4	37.6	34.6	34.8	34.6–37.6 (35.5 ± 1.2)
HDL	11.3	11.4	11.4	10.9	10.8	10.8–11.4 (11.1 ± 0.3)
HDW	11.3	11.6	11.2	10.8	10.6	10.6–11.6 (11.1 ± 0.4)
ED	4.0	4.1	4.1	3.8	4.4	3.8–4.4 (4.1 ± 0.2)
TD	2.4	2.3	2.2	2.2	2.4	2.2–2.4 (2.3 ± 0.1)
TED	1.4	1.4	1.4	1.5	1.4	1.4–1.5 (1.4 ± 0.1)
SNT	4.3	4.0	3.9	4.0	4.4	3.9–4.4 (4.1 ± 0.2)
IND	3.7	3.6	3.7	3.7	3.6	3.6–3.7 (3.6 ± 0.1)
IOD	3.2	3.4	3.4	3.4	3.4	3.2–3.4 (3.4 ± 0.1)
HDN	9.5	9.3	9.3	8.2	7.3	7.3–9.5 (8.7 ± 0.9)
RAD	7.2	7.1	6.9	6.0	5.8	5.8–7.2 (6.6 ± 0.6)
FTL	22.1	21.7	21.8	19.9	18.8	18.8–22.1 (20.8 ± 1.4)
TIB	15.1	15.4	15.6	14.0	13.5	13.5–15.6 (14.7 ± 0.9)
